# Downregulation of HP1α suppresses proliferation of cholangiocarcinoma by restoring SFRP1 expression

**DOI:** 10.18632/oncotarget.10371

**Published:** 2016-07-01

**Authors:** Wenlong Cheng, Li Tian, Bing Wang, Yongqiang Qi, Wenhua Huang, Hongbo Li, Yong-Jun Chen

**Affiliations:** ^1^ Department of Biliary-Pancreatic Surgery, Tongji Hospital, Tongji Medical College, Huazhong University of Science and Technology, Wuhan, Hubei Province, China; ^2^ Department of Wuhan Medical Care Center for Women and Children, Wuhan, Hubei Province, China; ^3^ Department of Hepatobiliary Surgery, The First Affiliated Hospital of Bengbu Medical College, Bengbu, Anhui Province, China

**Keywords:** HP1α, cholangiocarcinoma, proliferation, SFRP1, DNA methylation

## Abstract

Heterochromatin protein 1α (HP1α) is a gene that mediates chromatin conformation, gene silencing and cancer progression. However, little is known regarding the impact of HP1α in the pathogenesis of cholangiocarcinoma (CCA). In the present study, we demonstrate that HP1α is significantly upregulated in CCA tissues and cell lines, while downregulation of HP1α leads to suppression of cell proliferation. Then we find that downregulation of HP1α can decrease H3K9me3 enrichment and DNA methylation rate of secreted frizzled-related protein 1 (SFRP1) promoter, resulting in restoring the expression of SFRP1. Moreover, restoration of SFRP1 expression can suppress CCA cells proliferation. These results provide a mechanistic understanding of the role of HP1α in the pathogenesis of CCA and may offer a novel therapeutic target in this disease.

## INTRODUCTION

Cholangiocarcinoma (CCA) is a malignant tumor arising from bile duct epithelial cells. The prognosis of this disease is very poor, with a median survival of 6 to 12 months, and in recent decades a worldwide increase in the incidence of CCA has been reported [1]. Treatment for this disease is challenged by the limited response to chemoradiotherapy, and patients frequently present with advanced disease with limited options in regards to surgical resection [2]. As such, a deeper understanding of the molecular mechanisms of tumor initiation, progression, and metastasis of CCA is urgently required to develop effective therapeutic strategies.

Heterochromatin protein 1 (HP1) is a conserved non-histone chromosomal protein first discovered in Drosophila that localizes to centric and telomeric heterochromatin, participating in chromatin packaging and gene silencing [3, 4]. Three homologue isoforms of HP1 have been identified in mammals including HP1α, β and γ [5]. HP1α mainly localizes to heterochromatic regions of the genome, binding to trimethylated Lys9 of histone H3 (H3K9me3) [6]. Notably, recent evidences suggest that HP1α can also bind to euchromatin and dynamically participates in gene regulation [7–9], and in the context of cancer pathogenesis, a breast cancer study has shown correlations between alterations in HP1α protein levels and cancer progression [10]. Taken together, these results suggest that HP1α can contribute to tumor progression, although the molecular mechanisms require further clarification.

In the present study, we first performed a comprehensive analysis of HP1α expression profiles in CCA tissues and paired noncancerous bile duct tissues. We found that HP1α is significantly upregulated in CCA tissues and cell lines. Using overexpression and knockdown studies, we demonstrated that HP1α silencing can restore the expression of secreted frizzled-related protein 1 (SFRP1) via chromatin modifications which subsequently suppresses cell proliferation. These results provide a mechanistic understanding of the role of HP1α in the pathogenesis of CCA and may offer a novel therapeutic target in this disease.

## RESULTS

### HP1α expression is upregulated in CCA samples and associated with specific clinicopathological features

**Figure 1:**
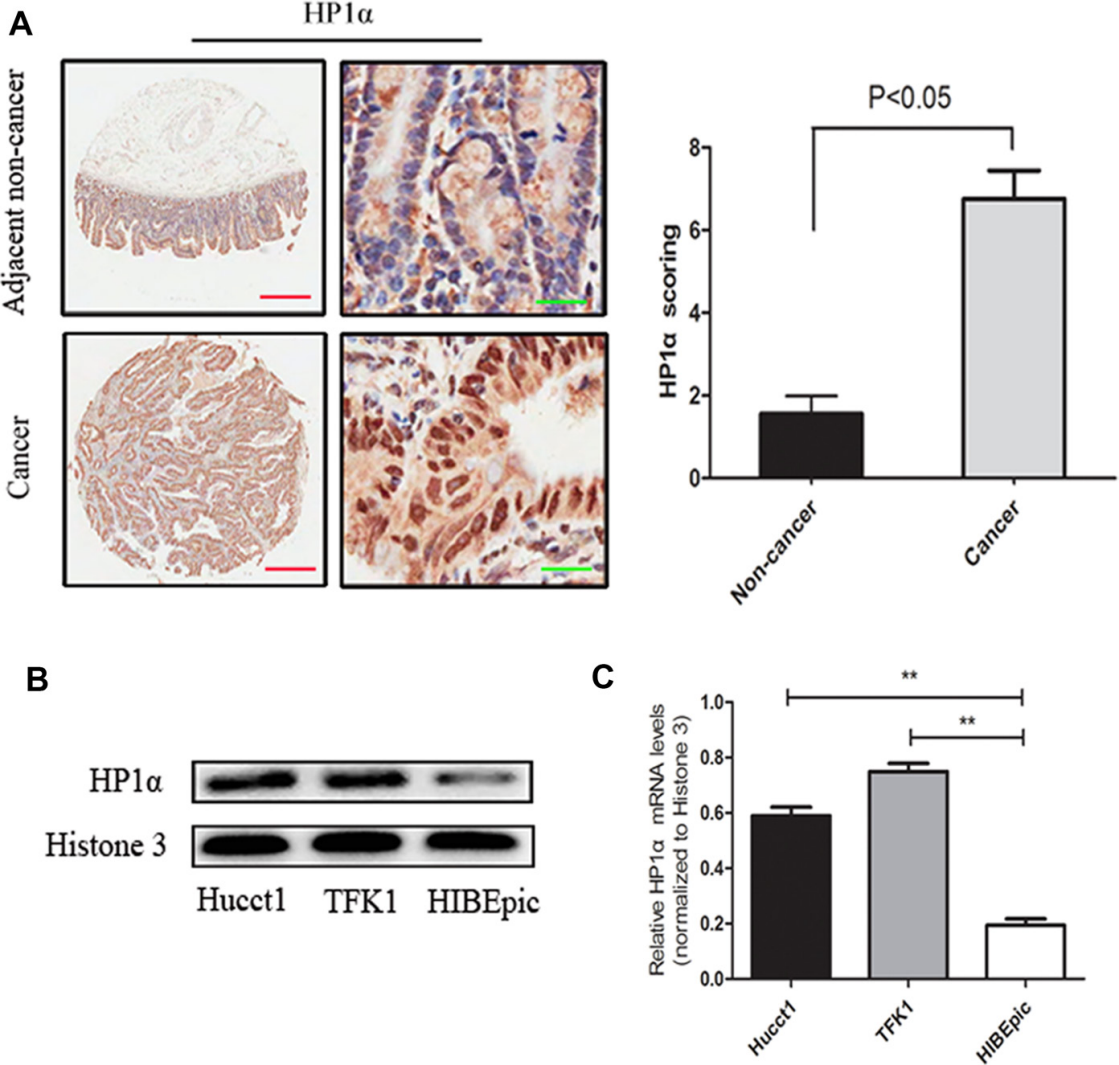
HP1α is upregulated in CCA tissues and cell lines (**A**) IHC analysis of HP1α expression in 40 CCA tissue samples and 22 paracancerous normal tissues. Representative images (left panel) and statistical analysis of HP1α expression (right panel) were taken. Scale bar, 500 μm (red line) or 25 μm (green line). (**B**) Western blot analysis of HP1α in Hucct1, TFK1 and HIBEpic cells. Histone 3 was used as an internal control. (**C**) Levels of HP1α in Hucct1, TFK1 and HIBEpic cells detected by quantitative real-time PCR analysis. Histone 3 was used as a internal control. **P < 0.01.

To investigate the clinical significance of HP1α expression, we first performed immunohistochemistry (IHC) analysis to evaluate its expression using a CCA tissue microarray (consisting of 27 CCA tissues and 9 paracancerous normal tissues) and 13 CCA samples with paired adjacent normal tissues. As shown in Figure 1A, the average expression level of HP1α was significantly higher in CCA tissues than in peritumoral tissues (stain index: non-cancer = 1.56 ± 0.43; cancer = 6.77 ± 0.68). We subsequently performed Western blot and quantitative real-time PCR to compare the expression of HP1α in CCA cell lines (Hucct1 and TFK1) versus the cholangiocyte cell line HIBEpic. Our results demonstrate that HP1α expression was significantly increased in these CCA cell lines (Figure 1B and 1C). We also investigated the correlation between HP1α expression and the clinicopathologic features of 40 CCA tissue samples. As shown in Table 1, reduced HP1α expression in tumor tissues was significantly associated with older age (> 60 years) and smaller tumor size (≤ 3 cm), but not with other parameters including sex, pathology grade and lymph node metastasis.

**Table 1:** Association between HP1α expression and clinicopathologic characteristics in 40 CCA tissues

Characteristics	Numbers of Patients	Low expression (IHC staining index: ≤ 6)	High expression (IHC staining index: > 6)	*P* value
Age (years)				
≤ 60	18	3	15	0.00*
> 60	22	16	6	
Sex				
Male	25	14	11	0.19
Female	15	5	10	
Tumor size (cm)				
≤ 3	24	16	8	0.00*
> 3	16	3	13	
Pathology grade				
Low (I + II)	28	13	15	1.00
High (III +IV)	12	6	6	
Lymph node metastasis				
Negative	24	13	11	0.35
Positive	16	6	10	

* Significant correlation.

### Downregulation of HP1α suppresses CCA proliferation *in vitro* and *in vivo*

**Figure 2:**
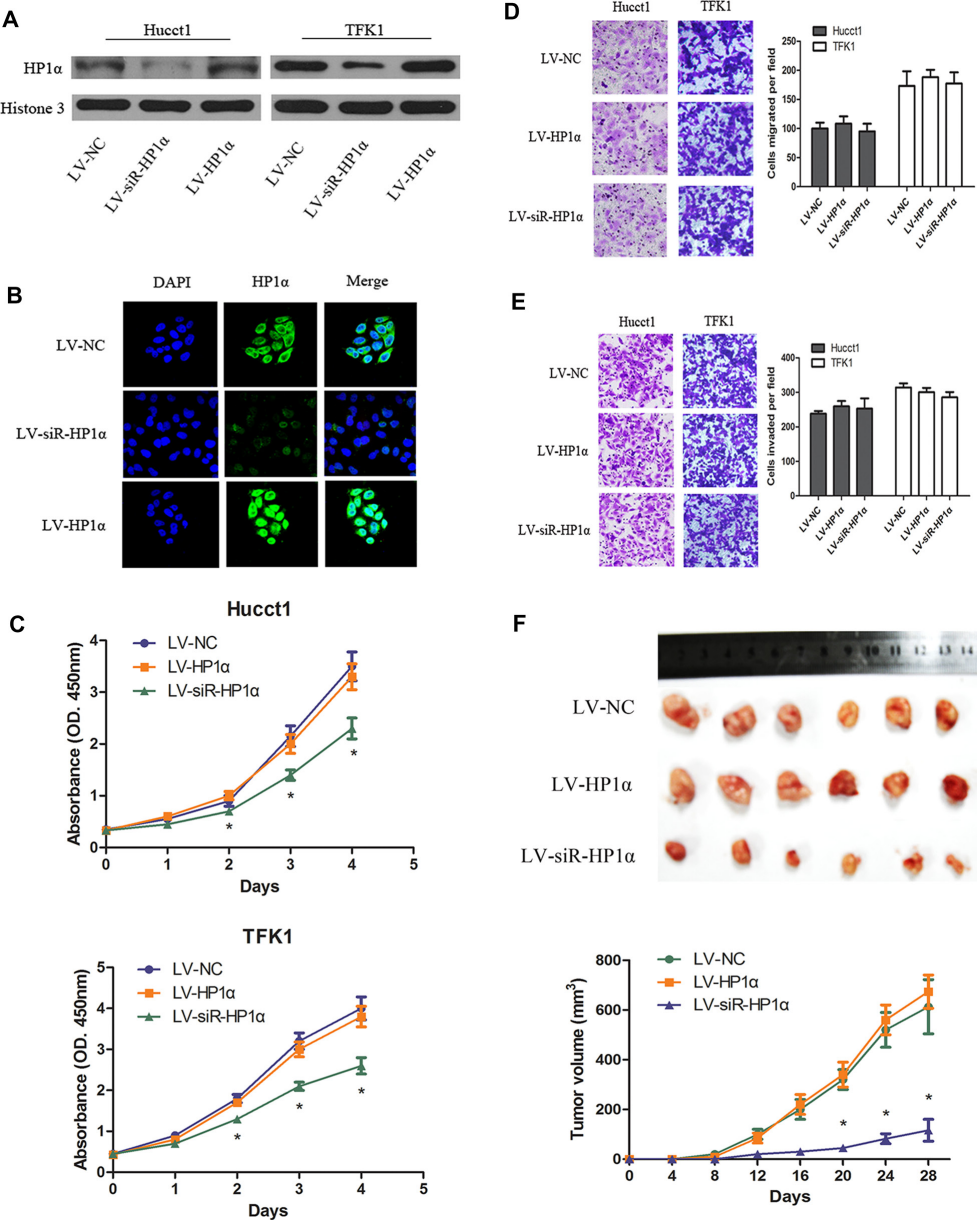
Downregulation of HP1α suppresses CCA proliferation *in vitro* and *in vivo* (**A**) Protein levels of HP1α following transfecting with lentiviral vectors. Histone 3 was used as a loading control. (**B**) Hucct1 cells were subjected to immunofluorescent staining of HP1α (green). DAPI was used to show the location of the nucleus (blue). Magnifications: ×400. (**C**) Indicated CCA cells were subjected to the CCK-8 assays for 4 days. Indicated cells were subjected to Transwell assays for migration (**D**) or for invasion (**E**). Representative images (left panel) and statistical comparison of indicated groups (right panel) were shown. Magnifications: ×200. (**F**) Representative photographs of tumors in nude mice (*N* = 6 per group) derived from LV-HP1α, LV-siR-HP1α and LV-NC Hucct1 cells. Statistical analysis of tumor volume in 3 groups every 4 days were taken in under panel. *P < 0.05.

To characterize the effects of HP1α in CCA cells, we stably altered HP1α expression through lentiviral-mediated transfection carrying HP1α, small interfering RNA (siRNA) targeting HP1α and negative control vectors, named LV-HP1α, LV-siR-HP1α and LV-NC, respectively. We performed Western blot and immunofluorescence (IF) analyses to confirm that HP1α was over-expressed in LV-HP1α cell lines and knocked down in LV-siR-HP1α cell lines (Figure 2A and 2B). We then assessed the effects of HP1α on cell proliferation, migration and invasion using the Cell Counting Kit-8 (CCK-8) assays and Transwell assays (Figure 2C, 2D and 2E). While downregulation of HP1α inhibited cell growth, no significant differences in migration and invasion were observed, as compared with the control cells. Interestingly, overexpression of HP1α had no significant impact on cell proliferation, migration or invasion, as compared to LV-NC cells. We thus assessed whether downregulation of HP1α could repress tumor growth *in vivo*. Figure 2F demonstrates that tumor growth in nude mice from LV-siR-HP1α group was significantly reduced as compared to the LV-NC group, and there were no significant differences in tumor growth between LV-HP1α group and LV-NC group. These results indicate that downregulation of HP1α could suppress CCA proliferation *in vitro* and *in vivo*.

### HP1α regulates chromatin modifications in CCA cells

**Figure 3:**
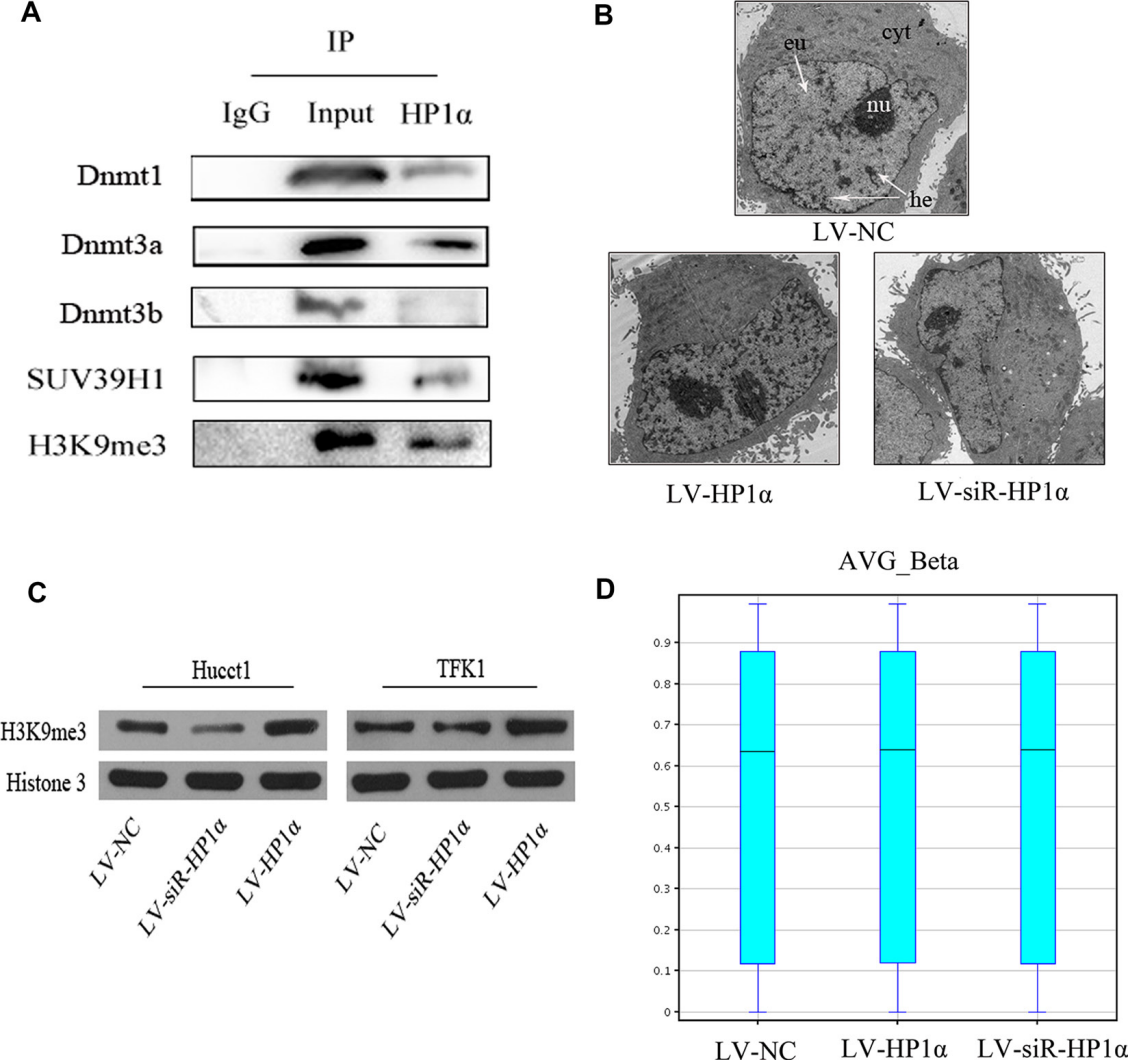
HP1α regulates chromatin modifications in CCA cells (**A**) Co-immunoprecipitation of Hucct1 cells with anti-HP1α antibodies, followed by Western blot. (**B**) Electron microscopy analysis of the distribution of heterochromatin in Hucct1 cells. Cyt: cytoplasm, nu: nucleus, eu: euchromatin, he: heterochromatin. Magnifications: ×1700. (**C**) Protein levels of H3K9me3 were detected by Western blot in CCA cells. Histone 3 was used as a loading control. (**D**) BoxWhisker Plot of Human 450 K Methylation microarray for evaluating the distribution of average CpGs methylation rate (AVG_Beta) with five statistic (the minimum value, the first quartile, the median, the third quartile and the maximum).

Previous studies reported that specific HP1α domains interact with selected partners to modulate the stability of heterochromatin [11]. The HP1α chromodomain (CD) binds to H3K9me3, and the HP1α chromoshadow-domain (CSD) interacts with the histone methyltransferase Suv39h and DNA methyltransferases (Dnmts). The model of H3K9me3/HP1α/SUV39H1/Dnmts complex was confirmed in many organisms such as Drosophila, mouse and human [12, 13]. We hypothesized that HP1α silences specific genes by modulating the organization of heterochromatin in CCA cells. To test this hypothesis, we performed co-immunoprecipitation (Co-IP) assays to confirm that HP1α interacts with H3K9me3, Dnmt1, Dnmt3a and SUV39H1 in Hucct1 cells (Figure 3A). We suspected that upregulation of HP1α could promote H3K9 methylation and DNA methylation and extend heterochromatin, while downregulation of HP1α could inhibit H3K9 methylation and DNA methylation thereby reducing heterochromatin.

We first evaluated the distribution of heterochromatin in different Hucct1 cell groups via electron microscopy (Figure 3B), and performed Western blot to assess the level of H3K9me3 (Figure 3C). Our reuslts demonstrate that both the distribution of heterochromatin and the level of H3K9me3 in LV-HP1α CCA cells were greater than those observed in LV-NC cells, while the distribution of heterochromatin and the level of H3K9me3 in LV-siR-HP1α CCA cells were less than those in LV-NC cells. Secondly, we analysed the global CpG methylation in Hucct1 cells using a Human Methylation 450 K microarray. Notably, there were no significant alterations in global CpG methyation across the three groups (Figure 3D). Taken together, while alteration of HP1α effects H3K9 methylation, there are no significant changes in global CpG methylation in CCA cells.

### HP1α regulates chromatin modifications from the level of gene such as SFRP1

The Human Methylation 450 K microarray evaluates 450,000 methylation sites across the genome, which covers 96% of all CpG islands. The chip is used to evaluate the average DNA methylation rate (AVG_Beta) among LV-HP1α, LV-siR-HP1α and LV-NC groups. When Delta_Beta [Delta_Beta = case(AVG_Beta) − control(AVG_Beta)] is greater than 0.17 or less than −0.17, the gene is defined as the differentially methylated gene.

**Figure 4:**
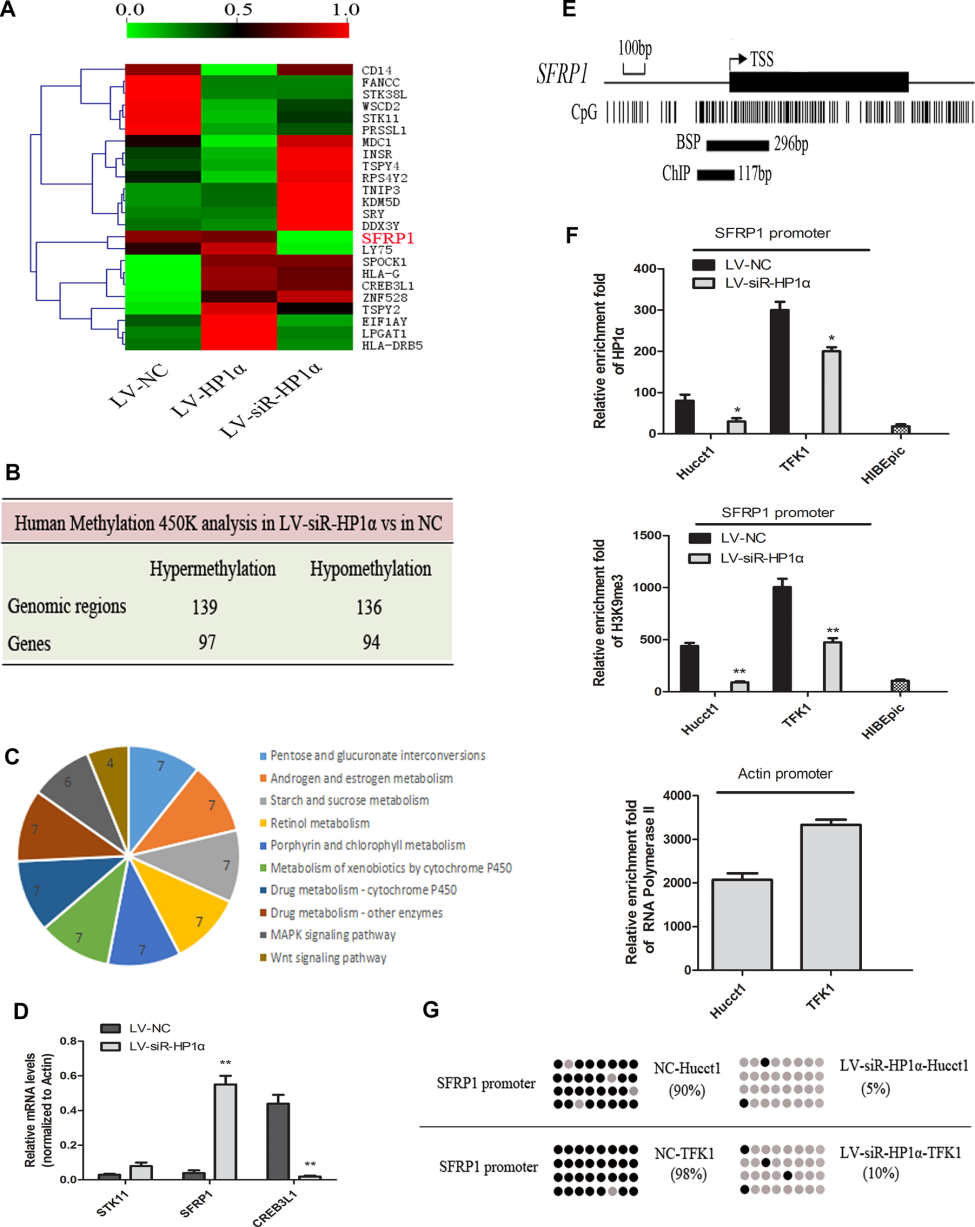
HP1α regulates chromatin modifications of SFRP1 promoter (**A**) Hierarchical clustering analysis on 24 most differentially methylated genes among LV-HP1α, LV-siR-HP1α and LV-NC cells. The AVG_Beta is represented in shades from green(0.0) to red(1.0). (**B**) Human Methylation 450 K analysis of the changed DNA methylation patterns between LV-siR-HP1α and LV-NC. (**C**) Ten pathways enriched with the most of differentially methylated genes between LV-siR-HP1α and LV-NC. (**D**) Levels of mRNA of STK11, SFRP1 and CREB3L1 were detected by quantitative real-time PCR analysis between LV-siR-HP1α and LV-NC. Actin was used as a internal control. (**E**) Graphic model of the CpG islands of SFRP1 promoter and primer amplification products of ChIP and BSP assays. (**F**) Indicated cells were subjected to ChIP assays for HP1α and H3K9me3 enrichment of SFRP1 promoter. IgG was served as a negative control. Relative enrichment fold = [%(ChIP/Input)]/[%(IgG/Input)]. Enrichment fold of RNA Polymerase II in the housekeeping gene Actin promoter was served as a positive control. (**G**) Indicated cells were subjected to BSP assays. Each row represented a single sequence analysed, and each dot was a single CpG site. Grey and black dots represented unmethylated and methylated CpGs, respectively. *P < 0.05, **P < 0.01.

Although there were no significant alterations in global CpG methylation among the three groups (as shown in Figure 3D), we have found special CpG sites were altered in some genes. A total of 275 genomic regions (191 genes) had significant changes in methylation patterns observed between LV-siR-HP1α Hucct1 and LV-NC Hucct1 cells, including 139 hypermethylated sites (97 genes) and 136 hypomethylated sites (94 genes) (Figure 4B). These 191 genes were classified into various cellular signaling pathways. Ten pathways enriched with the most of differentially methylated genes, largely relating to metabolism and proliferation, were summarized in Figure 4C. Then we performed hierarchical clustering on some of the most differentially methylated genes among the three groups (Figure 4A). We found that there was no correlation (either negative or positive) between alterations of HP1α and DNA methylation changes for these selected genes. We identified 19 genes that demonstrated the most differential methylation between LV-siR-HP1α and LV-NC. Detailed results of these genes were provided in Supplementary File S1. Three genes, including STK11, SFRP1 and CREB3L1, were involved in tumor-associated signaling pathway, and selected for further RT-PCR analysis. Our results (Figure 4D) demonstrate that downregulation of HP1α could significantly increase the expression of SFRP1 and decrease the expression of CREB3L1. However, there was no significant difference in the expression of STK11 between LV-siR-HP1α and LV-NC. As both SFRP1 and CREB3L1 could inhibit tumor proliferation [14, 15], reduced CREB3L1 expression could not inhibit CCA proliferation, we excluded CREB3L1 and STK11 from our analysis. Finally, we focused our attention on SFRP1, a known tumor suppressor gene. The promoter of SFRP1 was hypermethylated in LV-NC and LV-HP1α cells, and hypomethylated in LV-siR-HP1α cells. In order to investigate the correlation between HP1α expression and chromatin modifications to the SFRP1 promoter, we performed Chromatin immunoprecipitation (ChIP) and Bisulfite sequencing PCR (BSP). We researched the CpG islands (CpGs) of SFRP1 promoter and designed primers of ChIP and BSP assays (Figure 4E). ChIP analysis noted a reduced HP1α and H3K9me3 binding in the promoter region of SFRP1 in LV-siR-HP1α cells as compared to the control cell line (Figure 4F). Similarly, BSP analysis noted that the CpG methylation of the SFRP1 promoter was lower in LV-siR-HP1α cells as compared to controls (Figure 4G). In HIBEpic cells, the SFRP1 promoter was completely unmethylated, similar to LV-siR-HP1α cells. Collectively, these data suggest that downregulation of HP1α can reduce both H3K9 trimethylation and DNA methylation of target genes, including SFRP1, through decreasing activity of the H3K9me3/HP1α/SUV39H1/Dnmts complex.

### Downregulation of HP1α suppresses proliferation of CCA cells through the restoration of SFRP1 expression

**Figure 5:**
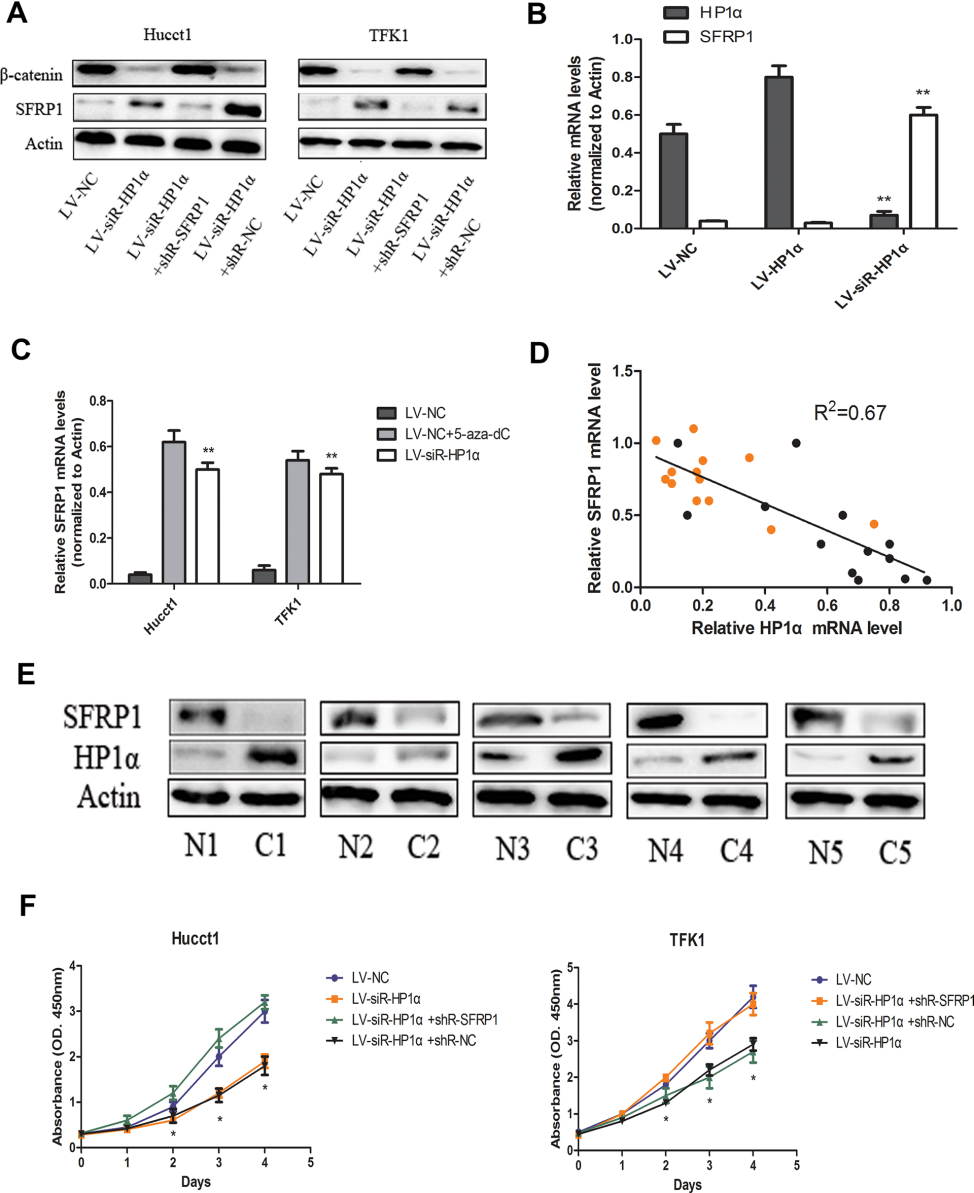
Downregulation of HP1α suppresses proliferation of CCA cells through the restoration of SFRP1 expression (**A**) Protein levels of SFRP1 and β-catenin were detected by Western blot in indicated CCA cells. Actin was used as a loading control. (**B**) Levels of SFRP1 and HP1α mRNA in tumors from nude mice using quantitative real-time PCR. Actin was used as a internal control. (**C**) Levels of SFRP1 mRNA in CCA cells were detected by quantitative real-time PCR analysis among LV-siR-HP1α group, LV-NC group and LV-NC+5-aza-dC group. Actin was used as a internal control. (**D**) The level of SFRP1 mRNA was negatively correlated with HP1α mRNA in CCA tissues and adjacent normal tissues. Black and yellow dots represented CCA and adjacent normal tissues, respectively. Statistical analysis was performed using Pearson's correlation coefficient. (R^2^ = 0.67, *P* < 0.01). (**E**) Protein levels of SFRP1 and HP1α were detected by Western blot in 5 paired human CCA samples and adjacent normal tissues. Actin was used as a loading control. (**F**) Indicated CCA cells were subjected to the CCK-8 assays for 4 days. *P < 0.05, **P < 0.01.

Previous studies have reported that SFRP1 promoter hypermethylation could serve as an epigenetic biomarker for cancer detection, progression and prognosis for breast and colorectal carcinomas [16, 17]. In the present study, we also detected SFRP1 promoter hypermethylation in LV-NC CCA cells (Figure 4G). In addition, we detected no SFRP1 expression in LV-NC CCA cells using Western blot (Figure 5A). To verify whether CpG methylation has any regulatory effect on SFRP1 expression, we performed qRT-PCR assays to compare SFRP1 levels in 5-aza-2′-deoxycytidine (5-aza-dC, a DNA methyltransferase inhibitor) treated and untreated CCA cells. As shown in Figure 5C, treatment with 5-aza-dC restored expression of SFRP1 in LV-NC CCA cells. The results indicate the expression of SFRP1 was affected by promoter methylation.

In last part, we revealed that downregulation of HP1α could reduce DNA methylation of the SFRP1 promoter (Figure 4G), then we performed Western blot to evaluate the differences of SFRP1 expression between LV-NC and LV-siR-HP1α cells (Figure 5A). We also verified the expression of HP1α and SFRP1 in tumors from nude mice (Figure 5B). The data suggests that downregulation of HP1α restores the expression of SFRP1 by demethylation of the promoter region.

In order to verify the correlation between HP1α and SFRP1 expression, we examined the mRNA expression of HP1α and SFRP1 in 13 paired CCA samples and adjacent normal tissues. We found a Pearson correlation coefficient of −0.8162 (*P* < 0.01), indicating a strong negative correlation between the expression of HP1α and SFRP1 in these samples (Figure 5D). In addition, 5 paired human CCA samples and adjacent normal tissues were also detected SFRP1 and HP1α expression (Figure 5E). The results that SFRP1 experssion of adjacent normal tissues, in which HP1α expression was reduced, were higher than CCA tissues supported our conclusions in some degree.

Accumulated evidences indicate that restoration of SFRP1 expression attenuates Wnt signaling and inhibits cell growth in certain tumor types [18]. To confirm the effects of HP1α on cell proliferation are mediated mainly via SFRP1 in CCA cells, we evaluated cell proliferation in LV-siR-HP1α cells following transfecting of an shRNA targeting SFRP1. Suppression of SFRP1 expression rescued the proliferative capacity of LV-siR-HP1α cells as compared to LV-NC cells (Figure 5A and 5F). These findings indicate that downregulation of HP1α can suppress proliferation of CCA cells through the restoration of SFRP1 expression.

It is well known that the Wnt signaling pathway is associated with many biologic processes such as differentiation, proliferation, and migration [19, 20]. SFRP1 can block Wnt signaling pathway to suppress tumor proliferation and metastasis [21]. Taken together, we hypothesize that the upregulation of SFRP1 via changes in HP1α expression suppresses proliferation of CCA cells through the Wnt signaling pathway. We performed Western blot in CCA cells to detect the levels of β-catenin, which is the best marker of activated Wnt signaling pathway. The results indicate that SFRP1 was upregulated and the β-catenin was downregulated in LV-siR-HP1α cells (Figure 5A). These results support our hypothesis that restoration of SFRP1 expression can supress CCA cells proliferation through inhibition of the Wnt signaling pathway.

## DISCUSSION

HP1α is an evolutionarily conserved non-histone chromosomal protein enriched in heterochromatin, and presents at specific euchromatic regions of the genome [7, 22]. It not only contributes to the stability of centromeres and telomeres, but also correlates with gene regulation and cancer progression [23]. Overexpression of HP1α can silence variegating genes [24, 25], while heterozygous mutation of HP1α results in less silencing of variegating genes [26]. Some studies have demonstrated a role of HP1 for regulating the biological behavior of cancer cells, but this topic has not been investigated in CCA to date. In the present study, we found that downregulation of HP1α suppresses cholangiocarcinoma proliferation through restoration of SFRP1 expression. We demonstrated that HP1α is significantly upregulated in CCA tissues, while reduced HP1α expression is significantly associated with older patients (> 60 years) and smaller tumor size (≤ 3 cm). Furthermore, we found that downregulation of HP1α could significantly suppress cell proliferation *in vitro* and *in vivo*. While a study in breast cancer demonstrated that HP1α is downregulated in highly invasive/metastatic cells relative to poorly invasive/non-metastatic cells. This discrepancy may be related to a cell-type specific effect.

**Figure 6:**
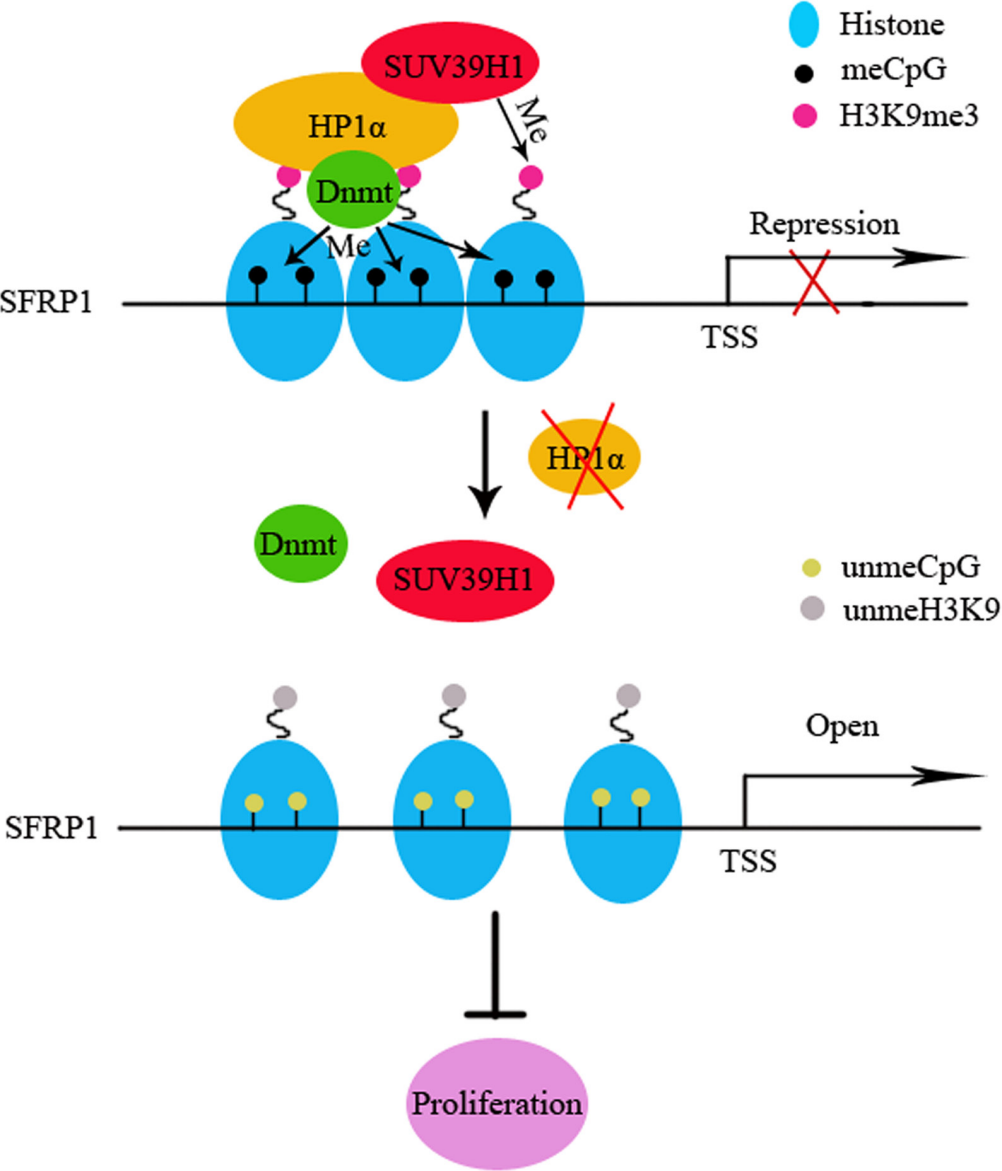
Schematic illustrations of the role of HP1α in CCA investigated in this study H3K9me3 creates a binding site for HP1α in euchromatic regions. HP1α recruits Dnmt1/3a to enhance cytosine methylation, resulting in transformation of euchromatin to silent heterochromatin and transcription silencing. In addition, HP1α recruits SUV39H1 to trimethylate H3K9 on adjacent histone to reinforce the silent heterochromatin state. When HP1α is down-regulated in CCA, the H3K9me3/HP1α/SUV39H1/Dnmt complex gets inactive, resulting in demethylation of H3K9 and CpGs of the SFRP1 promoter, then transcription opens.

To elucidate the mechanism by which reduced HP1α inhibits CCA proliferation, we focused on experiments about chromatin modifications. We demonstrated that downregulation of HP1α could reduce the distribution of heterochromatin in CCA cells. However, there were no significant alterations in global CpG methyation following changing HP1α expression. The reason for this phenomenon is that the specific CpG sites affected by HP1α are relatively few as compared to genome. We found that downregulation of HP1α could reduce H3K9me3 enrichment and CpG methylation rate of SFRP1 promoter, resulting in restoration of expression of this gene. Moreover, we demonstrated that activation of SFRP1 suppresses the proliferative capacity of CCA cells (Figure 6).

Several studies have highlighted an association between that HP1 expression levels and cancer progression. Two studies in breast cancer have demonstrated a causal role for HP1α regulating breast cancer progression through BRCA1 functions [10, 27]. Lee et al. [28] demonstrated that increased HP1β expression is associated with the poor prognosis in breast cancer, and HP1β is a potential predictive marker for PARP inhibitor therapy. Other studies have shown that HP1γ is upregulated in human colorectal cancer and promotes cell proliferation [29]. In addition, HP1γ expression is elevated in prostate cancer and represents a predictor of biochemical recurrence following radical prostatectomy [30].

While the importance of HP1 in cancer progression has been elucidated in previous studies, the specific contributions of HP1α expression towards the pathogenesis of CCA remain unknown. Our study represents the first characterization of HP1α in this cancer type, noting a significant overexpression as compared to normal adjacent tissue. Notably, downregulation of HP1α inhibits CCA cells proliferation through restoration of SFRP1 expression associated with histone modifications and DNA methylation changes. Our study broadens our understanding of the complex mechanisms underlying the pathogenesis of CCA and also suggests HP1 as a potential therapeutic target.

## MATERIALS AND METHODS

### Antibodies and tissue samples

Goat polyclonal anti-HP1α (Abcam, ab77256), rabbit polyclonal anti-H3K9me3 (Abcam, ab8898), rabbit polyclonal anti-SFRP1 (Abcam, ab4193), mouse monoclonal anti-RNA Polymerase II (Abcam, ab817), rabbit polyclonal anti-Dnmt1 (SantaCruz, sc-20701), rabbit polyclonal anti-Dnmt3a (SantaCruz, sc-20703), rabbit polyclonal anti-Dnmt3b (SantaCruz, sc-20704), rabbit polyclonal anti-SUV39H1 (Proteintech, 10574-1-AP), rabbit polyclonal anti-β-catenin (Proteintech, 51067-2-AP), rabbit polyclonal anti-Histone 3 (Proteintech, 17168-1-AP) and mouse monoclonal anti-β-actin (BOSTER, BM0626) were used as primary antibody. Normal IgG (Beyotime, A7007) was used as a control for co-immunoprecipitation. Rabbit anti-goat IgG-FITC (BOSTER, BA1110) were used as secondary antibody for immunofluorescence.

Thirteen paired human CCA and adjacent normal tissues samples were obtained from the Department of Biliary-Pancreatic Surgery, Tongji Hospital of Huazhong University of Science and Technology (HUST, Hubei, China). The collection of patient specimens was approved by the Ethic Committee of Tongji Hospital. Written informed consent was obtained from all patients.

### Cell culture and transfection

Human CCA cell lines (Hucct1 and TFK1) and cholangiocyte cell line HIBEpic which were conserved in our laboratory were maintained in RPMI 1640 supplemented with 10% fetal bovine serum (FBS) (Gibco, Grand Island, NY, USA), 100 U/mL penicillin and 100 μg/mL streptomycin in a humidified incubator containing 5% CO_2_ at 37°C.

Short hairpin RNA (shRNA) against the human SFRP1 gene (Target sequence: 5′-AGAAGAAGGACCTGAAGAA-3′) and small interfering RNA (siRNA) against the human HP1α gene (Target sequence: 5′-GGACAAGTGGAATATCTAC-3′) were purchased from RiboBio Co., Ltd, Guangzhou, China. Transient transfection was performed with Lipofectamine 2000 (Invitrogen, Camarillo, CA, USA) following the manufacturer's instructions. Transfected cells were incubated for 48 h, followed by cell harvesting and analysis.

Overexpression lentivirus vector of HP1α gene (GENE_ID: 23468, NM_012117), siRNA of HP1α lentivirus vector and negative control lentivirus (LV-NC, short for LV-siR-negative control) were constructed by Genechem Co., Ltd, Shanghai, China. The lentivirus vector construction was U6-MCS-Ubi-CHERRY-IRES-PURO. The lentiviruses were diluted in 0.3 ml (10^7^ TU/ml) complete medium containing polybrene in 25 ml cell culture flask and incubated at 37°C for 24 h. Then lentivirus medium was replaced with fresh 1640 medium and the cells were cultured for next 48 h.

### RNA extraction and quantitative real-time PCR

Total RNA was extracted from samples using Trizol reagent (Invitrogen) following the manufacturer's instruction. To quantify the mRNA levels, 400 ng of total RNA was subjected to first-strand cDNA synthesis using a PrimeScript RT Reagent kit (Takara, Dalian, China) according to the manufacturer's instructions. To quantify mRNA, 400 ng of total RNA was synthesized into cDNA using a PrimeScript RT Reagent kit (Takara, Dalian, China) according to the manufacturer's instructions. Real-time PCR was performed in a 10 μl reaction mixture with SYBR Premix Ex Taq (Takara) on the iQ5™ quantitative PCR detection system (Bio-Rad, Richmond, CA, USA) and the results were analyzed with IQ5 software. The primer sequences used for PCR include: HP1α-F: 5′-GGGAAAGAAAACCAAGCGGAC-3′ and HP1α-R: 5′-CACTTGTCCCTTAACCACGC-3′; SFRP1-F: 5′-GA GCCGGTCATGCAGTTCTT-3′ and SFRP1-R: 5′-CGT TGTCACAGGGAGGACAC-3′.

### Western blot

Tissues and cells were treated with cell lysis buffer for 30 min on ice. Protein extracts were cleared by centrifugation at 12,000 × g for 15 min at 4°C. A total of 50 μg of total proteins were separated on 12% polyacrylamide gel and transferred to PVDF membrane (Millipore). The membrane was blocked with 1% bovine serum albumin in TBST buffer (Tris Buffer Saline containing 0.1% Tween-20) for 2 h at room temperature, and subsequently incubated with primary antibodies described above over night at 4°C. Anti-rabbit, anti-mouse and anti-goat antibodies (BOSTER) were used as secondary antibodies for 2 h at 37°C. Protein bands were detected by enhanced chemiluminescence reagents ECL (Millipore, MA, USA) and the intensity of the bands was analyzed by Image J software (National Institute of Health, USA).

### Immunofluorescence

Hucct1 cells were plated onto glass coverslips in 6-well plates (5 × 10^4^ cells/well) and grown to approximately 50% confluence. Cells were fixed with 4% paraformaldehyde for 15 min at room temperature and permeabilized with 0.5% Triton X-100 in phosphate buffered saline (PBS) for 15 min at room temperature. Cells on coverslips were incubated with primary antibodies for 1 h at 37°C in the humidified box. Then cells were washed with PBS three times and incubated with fluorescent secondary antibodies for 45 min at 37°C in the humidified box. Cells were subsequently washed with PBS three times and incubated with DAPI (Beyotime). The samples were observed and photographed at 400×magnifications under a confocal laser scanning microscopy system (Leica).

### Co-immunoprecipitation

Co-immunoprecipitation of HP1α-associated proteins was performed using 2.5 μg anti-HP1α antibodies and 20 μl Protein A + G Agarose beads (Beyotime) according to manufacturer's instructions. 200 μg of protein extracts were added to the beads-antibody complex and mixed by rotation over night at 4°C. Each immunoprecipitated protein was detected by Western blot analysis using anti-Dnmt1, anti-Dnmt3a, anti-Dnmt3b, anti-SUV39H1 and anti-H3K9me3. Goat Normal IgG was used as a control for co-immunoprecipitation.

### Immunohistochemistry (IHC)

A CCA tissue microarray was purchased from Shanghai Outdo Biotech Co., Ltd and included 27 CCA tissues and 9 paracancerous normal tissues. IHC was performed on the tissue microarray and 13 paired CCA samples and adjacent normal tissues (previously described). All samples were deparaffinized, rehydrated through graded alcohol, washed with Tris-buffered saline, and processed using a streptavidin-biotin-peroxidase complex method. Antigen retrieval was performed by autoclaving the slides in 10 mM citric acid buffer. Samples were incubated with anti-HP1α antibodies diluted 1:700 for 4°C overnight. The corresponding secondary antibody was used for 30 min at 37°C. Slides were counterstained with hematoxylin before dehydration and mounting.

Immunohistochemical stains were scored semi-quantitatively according to the percentage and intensity of positive-staining cells. 0: < 5% positive cells; 1: 5% to 24% positive cells; 2: 25% to 49% positive cells; 3: 50% to 74% positive cells and 4: ≥ 75% positive cells. Intensity was scored as 0 for absence of staining, 1 for weak, 2 for moderate, and 3 for strong staining. Staining index = intensity × positive rate (absent, 0–1; mild, 2–4; moderate, 5–8; and strong, 9–12).

### Electron microscopy

Cells were collected and washed in 0.1 M sodium cacodylate, then fixed in a solution of 2% glutaraldehyde and 4% paraformaldehyde for 1 h at 4°C. Samples were treated postfixation with 1% OsO4 and 1% K_3_Fe(CN)_6_ for 40 min. After three washes with sodium cacodylate, samples were dehydrated with ethanol, treated with propylene oxide, and embedded in Epon. Ultrathin sections were prepared and examined at 200 kV under a Tecnai G^2^20 TWIN electron microscope (FEI, America).

### Chromatin immunoprecipitation (ChIP)

Histone modification ChIP assay was designed to calculate the percentage of loci that were precipitated in each treatment. Approximately 10^7^ cells were fixed with 1% formaldehyde to cross-link endogenous proteins and DNA. Samples of sonicated chromatin were immunoprecipitated with primary antibodies and protein A+G Agarose beads. IgG was served as a negative control. Immunoprecipitated DNA and input DNA were analyzed by quantitative real-time PCR using specific primers: SFRP1-F: 5′-G GTTGCAGTCAGCGGAGAT -3′ and SFRP1-R: 5′-G GAGCCTGGATCATACTTG C-3′; Actin-F: 5′-CCCTCCTCCTCTTCCTCAAT C T-3′ and Actin-R: 5′-AACGGCGCACGCTGATT-3′.

### Bisulfite sequencing PCR (BSP)

BSP primers were designed according to MethPrimer program on line (http://www.urogene.org/methprimer). Primer sequences for BSP were as following: BSP-SFRP1-F: 5′-TYGGGAGTTGATTGGTTG-3′ and BSP-SFRP1-R: 5′-CTTCCAAAAACCTCCR A A A A-3′. BSP reactions were performed using ddH_2_O, 10 × PCR buffer, dNTP mix, PCR primer, rTaq and bisulfite converted DNA samples in 25 μl final volume. PCR amplification was carried out using the following conditions: 94°C for 5 min, followed by 40 cycles of 94°C for 30 s, 58°C for 30 s, 72°C for 30 s. The PCR product was subcloned into the pMD19-T vector, and 10 clones from each group were randomly selected and sent for Sanger sequenced by Oebiotech Co (Shanghai, China).

### Cell proliferation assays

Cells were seeded at a density of 5 × 10^3^ cells per well in the 96-well plate containing 100 μl RPMI 1640 medium and 10% FBS. Cell Counting Kit-8 (CCK-8) (Dojindo, Tokyo, Japan) reagent was added at 48 h after seeding and incubated at 37°C for 1 h. The data of optical density (OD) value at 450 nm was measured by a microplate reader (Bio-Rad).

### Cell migration and invasion assays

Transwell assays were evaluated for migration (uncoated) and invasion (coated) activities of cells. A total of 1 × 10^5^ cells were resuspended in serum-free medium and dropped in the upper chamber, while 600 μl medium containing 10% FBS was placed in the lower well. After 36 h, cells that did not migrate or invade were removed using a cotton swab. Cells at the bottom of the membrane were fixed in 4% paraformaldehyde, stained with 1% crystal violet, and counted under a microscope.

### Tumorigenicity assays in nude mice

2 × 10^5^ Hucct1 cells in 200 μl PBS were subcutaneously injected into four-week-old male BALB/C nude mice, purchased from the Beijing HFK Bioscience Co., Ltd. Tumor growth was measured with calipers every 4 days and the tumor volumes were calculated using the formula: 0.5 × length × width^2^. All mice were sacrificed after 4 weeks. The study was approved by the Experimental Animal Ethics Committee of Tongji Medical College of Huazhong University of Science and Technology.

### Illumina 450 K methylation microarray

Following bisulfite treatment, the whole genome was amplified, enzymatically fragmented and hybridized to the 450 K Illumina Infinium Methylation BeadChip kits (Illumina, Inc., San Diego, USA). Following hybridization, allele specific single-base extension and staining were performed and then the BeadChips were imaged on Illumina BeadArray Reader. The image intensities were extracted using Illumina's BeadScan software (Illumina iScan scanner). Array data export processing and analysis were performed using Illumina GenomeStudio v2011.1 (Methylatioin Module v1.9.0) and the statistical computing package R 3.0.2. Data analyses including Differential Methylation analysis, Gene Ontology analysis and Pathway analysis were performed.

### Statistical analysis

All data were representative of three independent experiments and were presented as the mean ± standard deviation (SD). A two-sample *t*-test was performed to analyze two independent samples, whereas analysis of variance was conducted for comparison among groups. GraphPad Prism 5.0 (GraphPad Software) was used to calculate the *P*-value and a *P* < 0.05 was considered to be statistically significant.

## SUPPLEMENTARY MATERIALS




