# Supplementary material for: Downregulation of HP1α suppresses proliferation of cholangiocarcinoma by restoring SFRP1 expression
Source: Oncotarget. 2016 Jul 1;7(30):48107–19. doi: 10.18632/oncotarget.10371 (PMC5217004; doi:10.18632/oncotarget.10371)
Supplement: Supplementary file 1 [file oncotarget-07-48107-s001.pdf]

Downregulation of HP1α suppresses proliferation of cholangiocarcinoma by restoring SFRP1 expression

Supplementary Materials

Supplementary File S1: Detailed results of 19 genes including relevant disease and pathway.
See Supplementary_File_S1
